# Yixinjiedu Formula Attenuates Pressure Overload-Induced Cardiac Dysfunction by Suppressing Ferroptosis and Restoring Mitophagy via the PINK1/Parkin Axis

**DOI:** 10.3390/ph19030360

**Published:** 2026-02-25

**Authors:** Kang Xie, Haowen Zhuang, Xin Dong, Yulin Ouyang, Xin Liu, Zhongzheng Zhang, Mengyuan Wang, Jinhong Chen, Xinmeng Teng, Wei Wang, Chun Li, Junyan Wang

**Affiliations:** 1 School of Pharmaceutical Sciences, Guangzhou University of Chinese Medicine, Guangzhou 510006, China; 2 Guangdong Provincial Key Laboratory of Syndrome and Formula, School of Pharmaceutical Sciences, Guangzhou University of Chinese Medicine, Guangzhou 510006, China; 3 The First Affiliated Hospital, Guangzhou University of Chinese Medicine, Guangzhou 510405, China; 4 School of Basic Medical Sciences, Guangzhou University of Chinese Medicine, Guangzhou 510006, China; 5 State Key Laboratory of Traditional Chinese Medicine Syndrome, School of Pharmaceutical Sciences, Guangzhou University of Chinese Medicine, Guangzhou 510006, China; wangwei26960@126.com; 6 Guangdong Provincial Laboratory of Traditional Chinese Medicine, Hengqin, Zhuhai 519031, China

**Keywords:** Yixinjiedu formula, heart failure, ferroptosis, mitophagy, PINK1/Parkin pathway

## Abstract

**Background**: Pressure overload-induced heart failure (HF) involves cardiac remodeling, ferroptosis, and impaired mitophagy. Yixinjiedu formula (YXJDF), a traditional Chinese medicine, shows cardiovascular protective effects, but its underlying mechanisms remain largely unclear. This study aims to evaluate the cardioprotective effect of YXJDF in pressure overload-induced HF and explore its regulatory role in ferroptosis and mitophagy. **Methods**: A transverse aortic constriction (TAC) mouse model and angiotensin II-induced HL-1 cardiomyocytes were used to assess the therapeutic effects of YXJDF. Cardiac function, ferroptosis, and mitophagy were evaluated using histological, biochemical, molecular, and imaging analyses. Autophagic flux was assessed using lysosomal inhibition. Network pharmacology was applied to identify potential targets, while LC-MS/MS profiling and molecular docking were used to characterize major constituents of YXJDF and predict target interactions. **Results**: In TAC mice, YXJDF significantly improved cardiac function and attenuated myocardial hypertrophy and fibrosis. YXJDF suppressed ferroptotic injury, as evidenced by reduced lipid peroxidation, restoration of GPX4 and FTH1 expression, and normalization of antioxidant capacity. Mitophagy was restored, as indicated by increased PINK1 and Parkin expression, enhanced LC3-II accumulation, and reduced p62 and TOM20 levels, and as confirmed by autophagic flux analysis. Consistent protective effects on ferroptosis and mitophagy were observed in angiotensin II-induced cardiomyocytes. Network pharmacology analysis identified PINK1 as a key target, which was validated by in vivo and in vitro experiments. LC-MS/MS identified 20 major chemical constituents in YXJDF, and molecular docking showed strong binding affinity between several compounds (e.g., calycosin, salvianolic acid A) and PINK1. **Conclusions**: YXJDF ameliorates pressure overload-induced cardiac injury by restoring PINK1/Parkin-mediated mitophagy and suppressing ferroptosis. These findings reveal a multi-target mechanism underlying the therapeutic potential of YXJDF in HF.

## 1. Introduction

Heart failure (HF) is the final stage of many cardiovascular diseases and remains a major cause of morbidity and mortality worldwide. It is clinically characterized by dyspnea, fatigue, and fluid retention, with more than 64 million individuals affected globally [1,2]. The five-year survival rate is below 50%, comparable to that of certain malignant tumors, and the median survival time in advanced HF is only 12.2 months [3]. Although pharmacologic therapies such as ACE inhibitors, β-blockers, aldosterone antagonists, and angiotensin receptor-neprilysin inhibitors have become standard treatment options, these agents are often associated with adverse effects including bradycardia, hypotension, renal insufficiency, and gastrointestinal symptoms [4]. Thus, the development of safer and more effective treatment strategies remains a critical need.

Among the various causes of HF, sustained pressure overload, such as that resulting from hypertension or aortic stenosis, plays a key role, particularly in the development of HF with preserved ejection fraction [5]. The pathogenesis of pressure overload-induced HF is complex and multifactorial, involving not only structural and hemodynamic alterations but also maladaptive cellular responses [6,7]. Ferroptosis, an iron-dependent form of regulated cell death, has gained attention in recent years due to its involvement in cardiomyocyte injury, which is characterized by intracellular iron accumulation, lipid peroxidation, and depletion of antioxidants such as glutathione [8,9]. In cardiomyocytes, ferroptosis results in membrane disruption, mitochondrial damage, and contractile dysfunction [10]. Mitochondria play a central role in maintaining cardiac function by regulating energy production, redox balance, and calcium homeostasis [11]. They serve as both a source and a target of ferroptotic damage, as mitochondrial iron accumulation, excess reactive oxygen species (ROS) production, and impaired respiratory function jointly contribute to lipid peroxidation and compromise cellular energy metabolism [12]. Conversely, mitophagy, particularly the PTEN-induced putative kinase 1 (PINK1)/Parkin E3 ubiquitin ligase (Parkin)-mediated pathway, plays a critical role in mitochondrial quality control by selectively removing damaged mitochondria and limiting excessive ROS production [13]. Dysregulation of PINK1/Parkin-dependent mitophagy has been implicated in cardiac remodeling and HF progression. Impaired mitophagic flux has been shown to increase susceptibility to ferroptosis by amplifying oxidative injury [14]. These findings highlight the importance of targeting both ferroptosis and mitochondrial quality control mechanisms in the treatment of HF and suggest that therapeutic approaches capable of modulating multiple pathological processes may offer significant benefits.

Traditional Chinese medicine (TCM) formulations, composed of multiple herbal ingredients, may represent a promising option in complex diseases due to their multi-component and multi-target characteristics [15,16]. Unlike conventional single-target pharmaceuticals, TCM prescriptions are thought to exert synergistic effects on interconnected pathological processes, including inflammation, oxidative stress, metabolic imbalance, and organ dysfunction. In the context of cardiovascular disease, several classic TCM formulas have shown clinical efficacy in improving cardiac function and remodeling [17,18]. Yixinjiedu formula (YXJDF), also named Qishen granules, is a TCM prescription derived from classical cardio-protective principles, consisting of herbs such as *Astragalus camptoceras* Bunge, *Aconitum carmichaelii* Debeaux, and *Salvia miltiorrhiza* Bunge. It has been used in the treatment of chronic HF and coronary artery disease, with reported benefits in enhancing myocardial energy metabolism, reducing oxidative stress, and alleviating fibrosis [19,20,21]. However, the precise molecular mechanisms of YXJDF, particularly its effects on PINK1/Parkin-mediated mitophagy and ferroptosis in pressure overload-induced HF, remain largely undefined.

In this study, we investigated the cardioprotective effects of YXJDF using a murine transverse aortic constriction (TAC) model and an in vitro angiotensin II (Ang II)-stimulated HL-1 cardiomyocyte model. We focused on ferroptosis- and mitophagy-related processes by assessing cardiac function, mitochondrial integrity, autophagic flux, lipid peroxidation, and iron homeostasis. In parallel, network pharmacology, LC-MS/MS, and molecular docking were applied to identify potential molecular targets and active compounds underlying the therapeutic effects of YXJDF.

## 2. Results

### 2.1. YXJDF Attenuates Pressure Overload-Induced Cardiac Dysfunction and Remodeling in Mice

**Figure 1:**
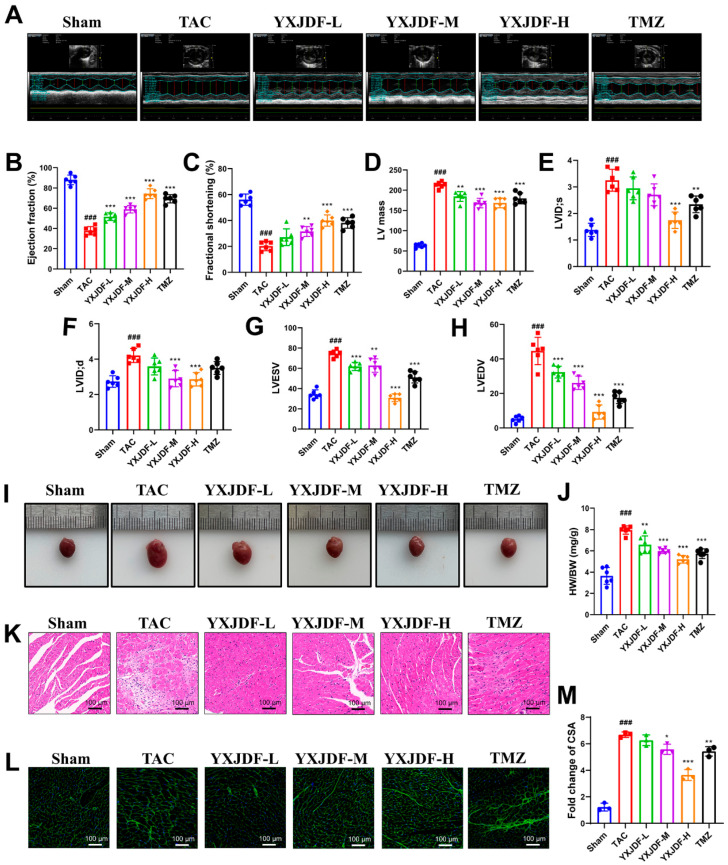
YXJDF attenuates pressure overload-induced cardiac dysfunction and remodeling induced by transverse aortic constriction (TAC). (**A**) Representative M-mode echocardiographic images from each group. (**B**–**H**) Quantitative analysis of cardiac function and structural parameters, including ejection fraction (%), fractional shortening (%), left ventricular mass (LV mass), left ventricular internal diameter at systole (LVID;s) and diastole (LVID;d), and left ventricular end-systolic and end-diastolic volumes (LVESV and LVEDV), as assessed by echocardiography (n = 6). (**I**) Representative gross morphology of harvested mouse hearts. (**J**) Heart weight to body weight ratio (HW/BW) as a normalized index of cardiac hypertrophy. (**K**) Hematoxylin and eosin (H&E) staining showing histological changes and inflammatory infiltration in cardiac tissue. (**L**) Wheat germ agglutinin (WGA) staining showing cardiomyocyte cross-sectional area (CSA). (**M**) Quantification of CSA from WGA-stained sections. Data are presented as mean ± standard deviation (SD). ^###^ *p* < 0.001 vs. Sham; * *p* < 0.05, ** *p* < 0.01, *** *p* < 0.001 vs. TAC. Abbreviations: Sham, sham-operated group; YXJDF, Yixinjiedu formula; TMZ, trimetazidine.

To investigate the cardioprotective effect of YXJDF against pressure overload-induced injury, we established a mouse model of TAC. Echocardiographic evaluation revealed that TAC surgery led to significant declines in ejection fraction and fractional shortening, accompanied by increased left ventricular internal diameters (LVIDs and LVIDd), LV mass, LVEDV, and LVESV, indicating marked cardiac dysfunction and hypertrophy. Treatment with YXJDF, particularly at medium and high doses, significantly improved EF and FS while alleviating ventricular dilatation and hypertrophy (Figure 1A–H).

Gross morphological observation showed apparent cardiac enlargement in TAC mice, which was mitigated by YXJDF intervention (Figure 1I). Consistently, HW/BW, a normalized index of cardiac hypertrophy, was significantly increased in TAC mice and was markedly reduced following YXJDF administration (Figure 1J). H&E staining further demonstrated extensive inflammatory infiltration and structural disorganization in the myocardium of TAC mice, both of which were alleviated following YXJDF administration (Figure 1K). Furthermore, WGA staining demonstrated a pronounced increase in cardiomyocyte CSA in the TAC group, which was markedly reduced by YXJDF treatment (Figure 1K), and quantitative analysis confirmed these changes (Figure 1L). These findings suggest that YXJDF effectively improves cardiac function and mitigates pathological remodeling in pressure overload-induced HF.

### 2.2. YXJDF Alleviates Myocardial Fibrosis in TAC-Induced Mice

**Figure 2:**
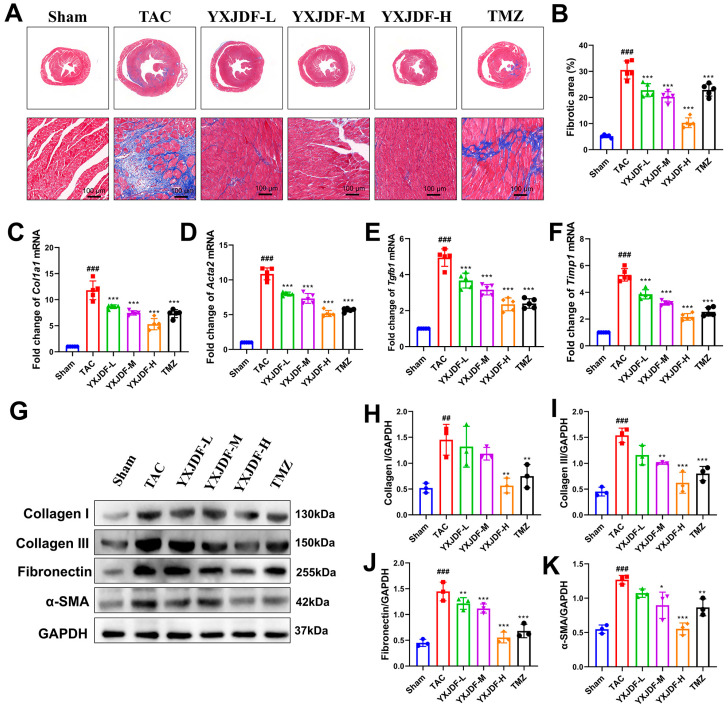
YXJDF attenuates myocardial fibrosis in TAC-induced mice. (**A**) Representative images of Masson’s trichrome staining in heart tissues from each group, showing collagen deposition (blue) and myocardial fibers (red) at low and high magnifications. (**B**) Quantification of fibrotic area percentage. (**C**–**F**) Quantitative real-time PCR (qRT-PCR) analysis of fibrosis-related genes, including *Col1a1*, *Acta2*, *Tgfb1*, and *Timp1*. (**G**) Western blot detection of fibrotic markers: Collagen I, Collagen III, Fibronectin, and α-smooth muscle actin (α-SMA). (**H**–**K**) Quantitative analysis of the corresponding protein expression normalized to GAPDH. Data are presented as mean ± SD (n = 3–5 biological replicates per group). ^##^ *p* < 0.01, ^###^ *p* < 0.001 vs. Sham; * *p* < 0.05, ** *p* < 0.01, *** *p* < 0.001 vs. TAC. Abbreviations: Sham, sham-operated group; TAC, transverse aortic constriction; YXJDF, Yixinjiedu formula; TMZ, trimetazidine.

To assess the effects of YXJDF on cardiac fibrosis, histological and molecular analyses were performed. Masson staining showed extensive interstitial and perivascular collagen deposition in the myocardial tissue of TAC mice, indicating severe cardiac fibrosis. Treatment with YXJDF, especially at medium and high doses, substantially reduced collagen accumulation compared to the untreated TAC group (Figure 2A). Quantitative analysis confirmed that the fibrotic area percentage was significantly decreased following YXJDF administration (Figure 2B). At the transcriptional level, qRT-PCR analysis demonstrated that TAC surgery significantly upregulated fibrogenic genes, including *Col1a1*, *Acta2*, *Tgfb1*, and *Timp1*, all of which were significantly suppressed by YXJDF (Figure 2C–F). At the protein level, Western blot analysis confirmed a pronounced increase in Collagen I, Collagen III, Fibronectin, and α-smooth muscle actin (α-SMA) in the TAC group. These profibrotic markers were notably downregulated by YXJDF in a dose-dependent manner (Figure 2G–K). These results indicate that YXJDF effectively alleviates myocardial fibrosis and pathological remodeling induced by pressure overload.

### 2.3. YXJDF Suppresses Myocardial Oxidative Stress and Ferroptosis in TAC-Induced Mice

**Figure 3:**
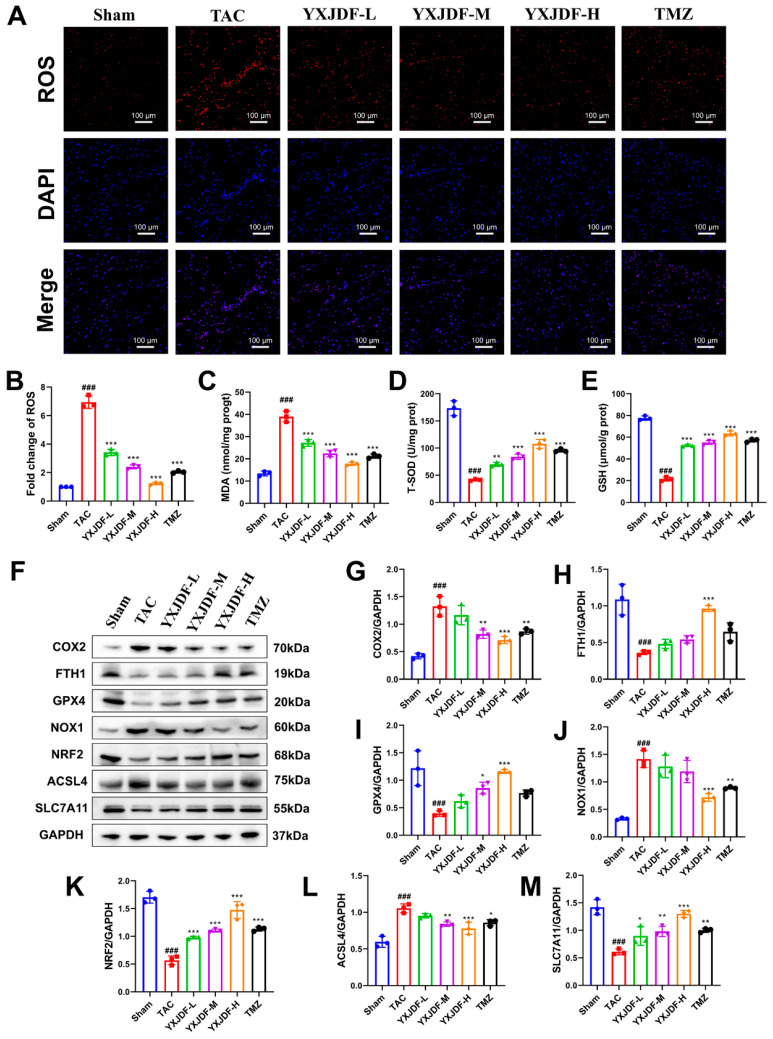
YXJDF alleviates oxidative stress and inhibits ferroptosis in the myocardium of TAC-induced mice. (**A**) Representative fluorescence images of cardiac tissue stained for reactive oxygen species (ROS) (red), with DAPI-labeled nuclei (blue) and merged overlays, showing elevated oxidative stress in the TAC group and its attenuation by YXJDF or TMZ treatment. (**B**) Quantification of ROS fluorescence intensity. (**C**–**E**) Biochemical measurement of oxidative stress indicators in myocardial tissue: MDA content, T-SOD activity, and GSH levels. (**F**) Representative Western blot bands for oxidative stress and ferroptosis-related proteins, including cyclooxygenase-2 (COX2), ferritin heavy chain 1 (FTH1), glutathione peroxidase 4 (GPX4), NADPH oxidase 1 (NOX1), nuclear factor erythroid 2–related factor 2 (NRF2), acyl-CoA synthetase long-chain family member 4 (ACSL4), and solute carrier family 7 member 11 (SLC7A11). GAPDH was used as a loading control. (**G**–**M**) Densitometric quantification of each ferroptosis marker normalized to GAPDH. Data are presented as mean ± SD (n = 3 biological replicates per group). ^###^ *p* < 0.001 vs. Sham; * *p* < 0.05, ** *p* < 0.01, *** *p* < 0.001 vs. TAC. Abbreviations: Sham, sham-operated group; TAC, transverse aortic constriction; YXJDF, Yixinjiedu formula; TMZ, trimetazidine.

To evaluate the antioxidant effects of YXJDF, ROS levels in myocardial tissue were first assessed by fluorescence staining. Compared with the sham group, ROS production was markedly increased in TAC mice, while treatment with YXJDF significantly reduced ROS accumulation in a dose-dependent manner (Figure 3A,B). Consistent with these molecular changes, MDA content was elevated, while T-SOD and GSH levels were decreased in TAC hearts. These abnormalities were significantly improved by YXJDF treatment (Figure 3C–E). Given the close association between oxidative stress and ferroptosis, we next examined the expression of ferroptosis-related markers. Cyclooxygenase-2 (COX2) and NADPH oxidase 1 (NOX1), which are involved in both oxidative stress and ferroptosis, were significantly elevated in TAC mice and downregulated upon YXJDF treatment. Western blot analysis further showed that TAC markedly increased the expression of other pro-ferroptotic proteins, such as acyl-CoA synthetase long-chain family member 4 (ACSL4), while reducing the levels of ferroptosis inhibitors, including ferritin heavy chain 1 (FTH1), glutathione peroxidase 4 (GPX4), nuclear factor erythroid 2–related factor 2 (NRF2), and solute carrier family 7 member 11 (SLC7A11). YXJDF administration reversed these alterations, suggesting suppression of ferroptotic activity (Figure 3F–M). Together, these results demonstrate that YXJDF alleviates oxidative stress and inhibits ferroptosis in the heart under conditions of pressure overload.

### 2.4. YXJDF-Containing Serum Protects HL-1 Cardiomyocytes from Ang II-Induced Injury by Improving Mitochondrial Function and Suppressing Ferroptosis

**Figure 4:**
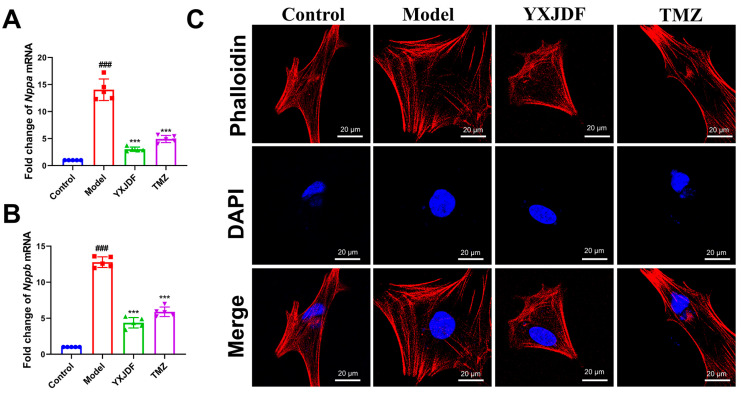
YXJDF-containing serum alleviates cardiomyocyte injury and cytoskeletal disruption induced by angiotensin II (Ang II). (**A**,**B**) Relative mRNA expression levels of *Nppa* and *Nppb* in HL-1 cardiomyocytes were detected by qRT-PCR after treatment with Ang II, with or without YXJDF-containing serum or trimetazidine (TMZ). (**C**) Representative images of phalloidin staining (red) and DAPI nuclear staining (blue) in each group, showing cytoskeletal integrity and cellular morphology (scale bar = 50 μm). Data are presented as mean ± SD (n = 5 biological replicates per group). ^###^ *p* < 0.001 vs. control; *** *p* < 0.001 vs. model. Abbreviations: YXJDF, serum obtained from rats treated with QiShen granule; TMZ, trimetazidine.

To further validate the cellular protective effects of YXJDF, HL-1 cardiomyocytes were stimulated with Ang II in vitro and treated with serum obtained from YXJDF-treated rats. Quantitative PCR analysis showed that Ang II significantly upregulated the mRNA expression of *Nppa* and *Nppb*, indicating cardiomyocyte stress and injury. YXJDF-containing serum markedly reduced the expression levels of both markers (Figure 4A,B). Phalloidin staining revealed substantial cytoskeletal disruption in the model group, while YXJDF treatment preserved filament structure and improved cellular morphology (Figure 4C).

**Figure 5:**
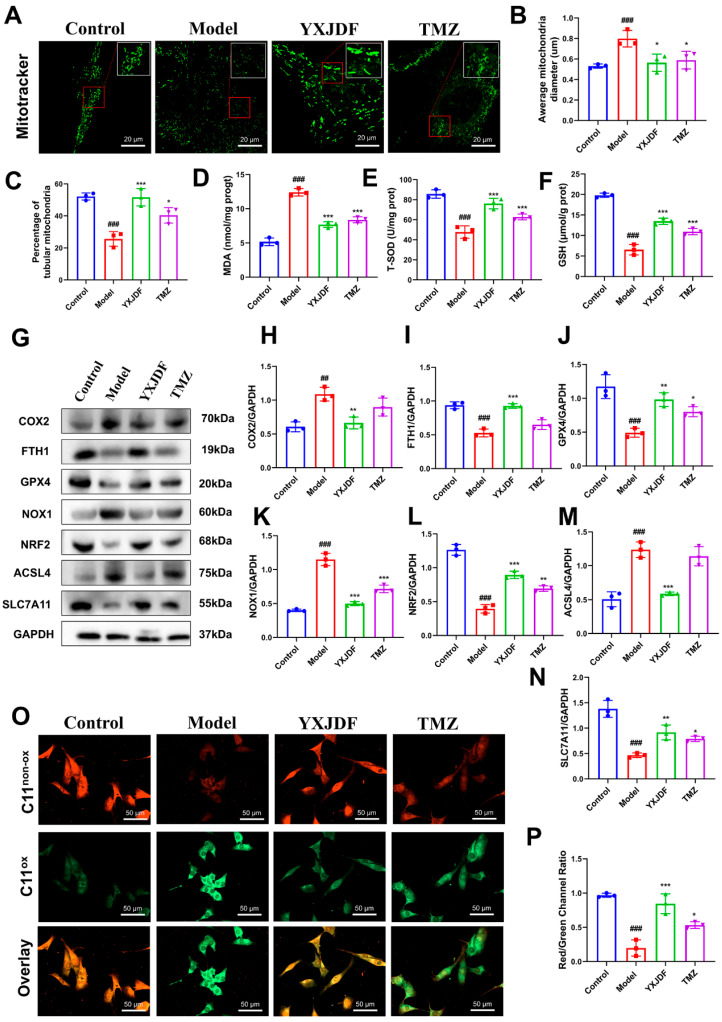
YXJDF-containing serum protects HL-1 cardiomyocytes from Ang II-induced oxidative stress and ferroptosis. (**A**) MitoTracker staining showed mitochondrial morphology in HL-1 cells. Scale bar = 20 μm. (**B**,**C**) Quantitative analysis of mitochondrial morphology, including average mitochondrial diameter and percentage of tubular mitochondria, based on MitoTracker-stained images. (**D**–**F**) Biochemical assays showing levels of intracellular malondialdehyde (MDA), reduced glutathione (GSH), and total superoxide dismutase (T-SOD) activity in HL-1 cells. (**G**) Western blot analysis of ferroptosis-related proteins, including cyclooxygenase-2 (COX2) and NADPH oxidase 1 (NOX1) (shared markers of oxidative stress and ferroptosis), the pro-ferroptotic enzyme acyl-CoA synthetase long-chain family member 4 (ACSL4), and anti-ferroptotic proteins ferritin heavy chain 1 (FTH1), glutathione peroxidase 4 (GPX4), nuclear factor erythroid 2–related factor 2 (NRF2), and solute carrier family 7 member 11 (SLC7A11). (**H**–**N**) Quantification of protein expression normalized to GAPDH. (**O**) C11-BODIPY 581/591 staining (a lipid peroxidation–sensitive fluorescent probe) showing lipid peroxidation in HL-1 cardiomyocytes. (**P**) Quantitative analysis presented as the green/red fluorescence ratio. Data are presented as mean ± SD (n = 3 biological replicates per group). ^##^ *p* < 0.01, ^###^ *p* < 0.001 vs. control; * *p* < 0.05, ** *p* < 0.01, *** *p* < 0.001 vs. model. Abbreviations: YXJDF, serum obtained from rats treated with QiShen granule; TMZ, trimetazidine.

Mitochondria play a pivotal role in cardiomyocyte survival and energy metabolism, and mitochondrial dysfunction is a hallmark of pressure overload-induced myocardial injury. To assess mitochondrial health, MitoTracker staining was performed. Ang II induced severe mitochondrial fragmentation and reduced mitochondrial fluorescence intensity, indicating depolarization of the mitochondrial membrane. These alterations were reversed by YXJDF treatment, suggesting improved mitochondrial integrity and membrane potential (Figure 5A–C). Moreover, biochemical assays revealed that oxidative stress was markedly elevated after Ang II exposure, as evidenced by reduced intracellular levels of GSH and T-SOD and elevated MDA levels. Treatment with YXJDF-containing serum significantly alleviated these abnormalities (Figure 5D–F). At the molecular level, Ang II induced upregulation of COX2 and NOX1, which are implicated in both oxidative stress and ferroptosis, along with increased ACSL4 and decreased expression of classic ferroptosis inhibitors such as FTH1, GPX4, NRF2, and SLC7A11. These dysregulated protein levels were effectively reversed by YXJDF-containing serum (Figure 5G–N). In addition, lipid peroxidation was directly evaluated using the ferroptosis-specific probe C11-BODIPY 581/591. Ang II stimulation markedly increased C11-BODIPY oxidation in HL-1 cardiomyocytes, as indicated by an elevated green/red fluorescence ratio, whereas YXJDF-containing serum significantly attenuated this lipid peroxidation response (Figure 5O). The trend of C11-BODIPY oxidation was consistent with the expression pattern of ACSL4 observed by Western blot analysis.

Collectively, these findings indicate that YXJDF protects HL-1 cardiomyocytes from Ang II-induced injury by preserving mitochondrial integrity, reducing oxidative stress, and suppressing ferroptosis.

### 2.5. Network Pharmacology Analysis Reveals PINK1 as a Potential Core Target of YXJDF in HF

**Figure 6:**
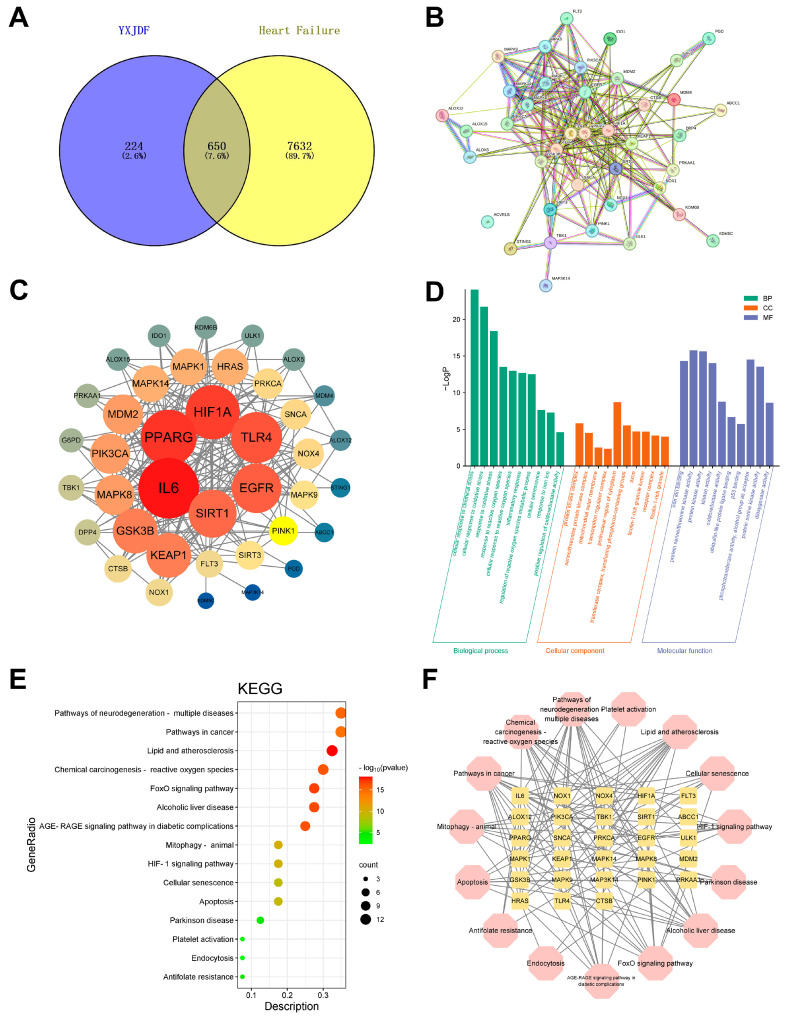
Network pharmacology analysis reveals potential therapeutic targets and pathways of YXJDF in heart failure (HF). (**A**) Venn diagram showing 650 overlapping targets between YXJDF-related and HF-related genes. (**B**) Protein–protein interaction (PPI) network constructed from the 650 intersecting genes using the Search Tool for the Retrieval of Interacting Genes/Proteins (STRING) database. (**C**) Core target network identified by topological analysis using CytoNCA plugin (version 2.1.6) in Cytoscape (version 3.9.1; https://cytoscape.org/) (network visualization software); node size and color intensity represent degree value. (**D**) Gene Ontology (GO) enrichment analysis results, including biological process (BP), cellular component (CC), and molecular function (MF) categories. (**E**) Kyoto Encyclopedia of Genes and Genomes (KEGG) pathway enrichment analysis showing the top 15 significantly enriched pathways. (**F**) Network visualization of core targets and their associated KEGG pathways. Data were analyzed and visualized using Cytoscape, STRING, Metascape (a web-based enrichment analysis platform), and bioinformatics platforms.

To explore the potential mechanisms of YXJDF against heart failure, network pharmacology analysis was performed. A total of 874 putative targets related to active compounds in YXJDF were obtained from public databases. These were intersected with 8282 heart failure-related genes retrieved from GeneCards and OMIM databases. A total of 650 overlapping genes were identified as potential therapeutic targets of YXJDF in the context of HF (Figure 6A). These intersected targets were imported into Cytoscape to construct a PPI network, which was visualized using Cytoscape (Figure 6B). The resulting network revealed dense interactions, suggesting that YXJDF regulates multiple interrelated biological processes. To identify core regulatory genes within this network, topological analysis was conducted using the CytoNCA plugin in Cytoscape. Key hub genes such as IL6, PPARG, SIRT1, EGFR, as well as PINK1, were identified based on degree centrality and other topological parameters (Figure 6C).

Further functional enrichment analysis of the 650 overlapping genes was conducted using GO and KEGG databases. Gene Ontology analysis identified a total of 156 enriched GO terms. Representative biological processes included response to oxidative stress, response to iron ions, inflammatory response, and apoptotic signaling. In the cellular component category, significant enrichment was observed in the mitochondrial outer membrane, macromolecular complex, and cytosol. For molecular function, key terms included oxidoreductase activity, protein kinase binding, and cytokine activity (Figure 6D). Together, these results suggest that YXJDF may exert therapeutic effects through regulating mitochondrial function, oxidative stress, and iron metabolism. KEGG pathway enrichment analysis revealed 167 significantly enriched pathways among the YXJDF-HF overlapping targets. These pathways were primarily related to lipid metabolism, mitochondrial dynamics, apoptosis, and cellular senescence (Figure 6E). Combined with results from the PPI network and GO enrichment, this finding suggests that PINK1-mediated mitophagy, along with ROS regulation, oxidative stress response, and iron ion homeostasis, may play key roles in the therapeutic mechanisms of YXJDF. This hypothesis was further supported by the network visualization of core targets and enriched pathways (Figure 6F).

### 2.6. YXJDF Activates the PINK1/Parkin-Mediated Mitophagy Pathway in Failing Myocardium and HL-1 Cardiomyocytes

Based on network pharmacology and enrichment analysis, PINK1 was identified as a potential key target of YXJDF in the treatment of HF. Parkin is functionally coupled to PINK1 in the canonical mitophagy pathway; both proteins were evaluated in subsequent in vivo and in vitro validation experiments. We also examined the expression of additional mitophagy-related markers, including Beclin-1, p62, microtubule-associated protein 1 light chain 3 (LC3), translocase of outer mitochondrial membrane 20 (TOM20), and autophagy-related protein 16-like 1 (ATG16L1), to comprehensively assess the effect of YXJDF on mitophagic activity.

**Figure 7:**
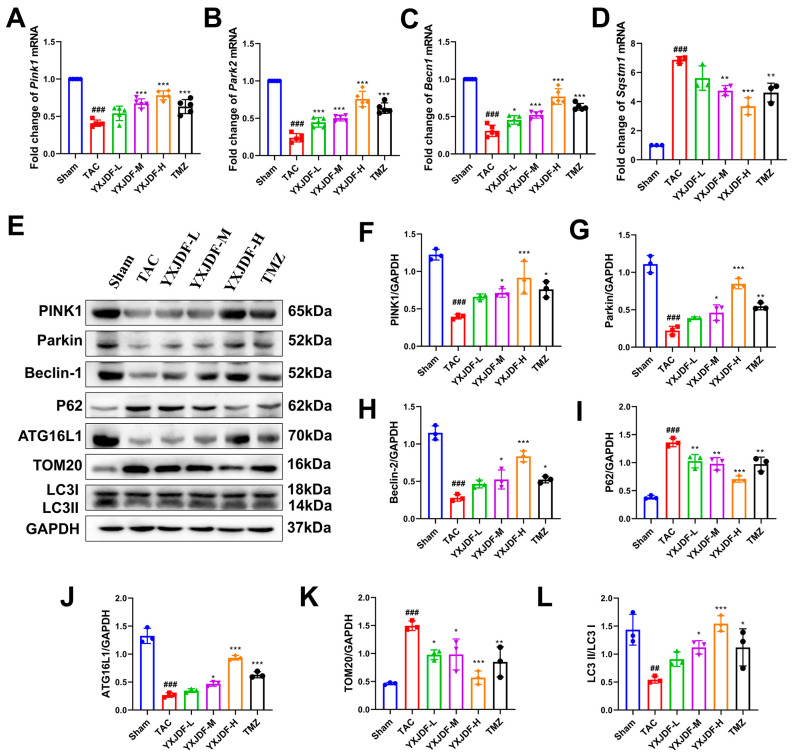
YXJDF activates the PTEN-induced putative kinase 1 (PINK1)/Parkin-mediated mitophagy pathway in TAC-induced failing hearts. (**A**–**D**) Quantitative real-time PCR (qRT-PCR) analysis of mitophagy-related genes, including *Pink1*, *Park2*, *Becn1*, and *Sqstm1* in myocardial tissue from each group. (**E**) Representative Western blot images showing protein expression of PINK1, Parkin, Beclin-1, p62, autophagy-related protein 16-like 1 (ATG16L1), translocase of outer mitochondrial membrane 20 (TOM20), microtubule-associated protein 1 light chain 3 (LC3)-I, and LC3-II in cardiac tissue. (**F**–**L**) Densitometric quantification of Western blot results for PINK1 (**F**), Parkin (**G**), Beclin-1 (**H**), p62 (**I**), ATG16L1 (**J**), TOM20 (**K**), and LC3-II/LC3-I ratio (**L**). Data are expressed as mean ± SD (n = 3–5 biological replicates). ^##^ *p* < 0.01, ^###^ *p* < 0.001 vs. Sham; * *p* < 0.05, ** *p* < 0.01, *** *p* < 0.001 vs. TAC. Abbreviations: Sham, sham-operated group; TAC, transverse aortic constriction; YXJDF, Yixinjiedu formula; TMZ, trimetazidine.

In vivo, qRT-PCR analysis revealed that YXJDF increased the transcription of *Pink1*, *Park2*, and *Becn1*, and decreased *Sqstm1* expression in a dose-dependent manner (Figure 7A–D). These findings were consistent with Western blot analysis, which showed that TAC significantly downregulated the expression of PINK1, Parkin, Beclin-1, ATG16L1, and the LC3-II/LC3-I ratio, while increasing the expression of TOM20 and p62 (Figure 7E–L). Notably, YXJDF treatment reversed these alterations, especially in the medium- and high-dose groups.

**Figure 8:**
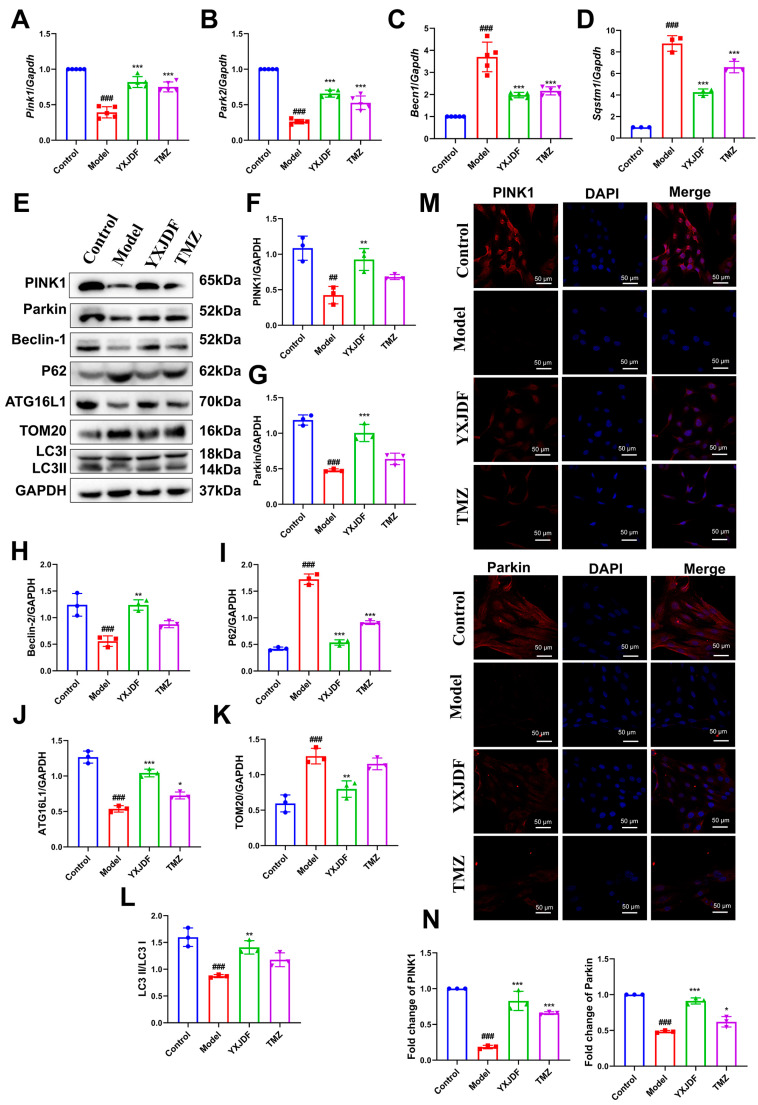
YXJDF-containing serum activates the PTEN-induced putative kinase 1 (PINK1)/Parkin-mediated mitophagy pathway in angiotensin II (Ang II)-treated HL-1 cardiomyocytes. (**A**–**D**) Quantitative real-time PCR (qRT-PCR) analysis of *Pink1*, *Park2*, *Becn1*, and *Sqstm1* mRNA expression in each group. (**E**) Representative Western blot bands showing protein expression of PINK1, Parkin, Beclin-1, p62, autophagy-related protein 16-like 1 (ATG16L1), translocase of outer mitochondrial membrane 20 (TOM20), and microtubule-associated protein 1 light chain 3 (LC3)-I, and LC3-II. (**F**–**L**) Quantification of PINK1 (**F**), Parkin (**G**), Beclin-1 (**H**), p62 (**I**), ATG16L1 (**J**), TOM20 (**K**), and LC3-II/LC3-I ratio (**L**) based on Western blot. (**M**,**N**) Representative immunofluorescence images (M) and quantitative analysis (**N**) of PINK1 and Parkin expression and subcellular localization in HL-1 cells. Nuclei were stained with 4′,6-diamidino-2-phenylindole (DAPI, blue), and target proteins were labeled in red. Data are expressed as mean ± SD (n = 3–5 biological replicates per group). ^##^ *p* < 0.01, ^###^ *p* < 0.001 vs. Control; * *p* < 0.05, ** *p* < 0.01, *** *p* < 0.001 vs. Model. Abbreviations: YXJDF, serum obtained from rats treated with QiShen granule; TMZ, trimetazidine.

In vitro, consistent transcriptional patterns were observed in HL-1 cardiomyocytes. Ang II exposure markedly suppressed *Pink1*, *Park2*, and *Becn1* expression while upregulating *Sqstm1*. YXJDF-containing serum effectively normalized these gene expression levels (Figure 8A–D). At the protein level, YXJDF restored mitophagy-associated markers, evidenced by increased levels of PINK1, Parkin, Beclin-1, ATG16L1, and LC3-II/LC3-I, and reduced p62 and TOM20 expression (Figure 8E–L). Furthermore, immunofluorescence staining confirmed the restoration of PINK1 and Parkin protein localization upon YXJDF treatment (Figure 8A–D). Taken together, these data provide strong evidence that YXJDF enhances PINK1/Parkin-mediated mitophagy, thereby contributing to mitochondrial quality control under conditions of pressure overload and Ang II-induced cellular stress.

To further determine whether the YXJDF-induced changes in mitophagy-related markers reflected a genuine increase in mitophagic flux rather than altered lysosomal degradation, autophagic flux analysis was performed using the lysosomal inhibitor BafA1. As shown in Supplementary Figure S1A–D, BafA1 treatment led to pronounced accumulation of LC3-II and p62 in both control and YXJDF-treated cells, confirming effective lysosomal blockade. Notably, under BafA1 co-treatment, YXJDF resulted in a greater accumulation of LC3-II compared with BafA1 alone, while the YXJDF-induced reduction in TOM20 was abolished. These findings indicate that YXJDF promotes mitochondrial turnover through an increase in mitophagic flux rather than by inhibiting lysosomal degradation. Together, these flux analyses provide additional evidence that YXJDF restores functional PINK1/Parkin-dependent mitophagy under Ang II–induced stress conditions.

### 2.7. Park2 Knockdown Weakens the Protective Effects of YXJDF on Mitophagy and Ferroptosis

**Figure 9:**
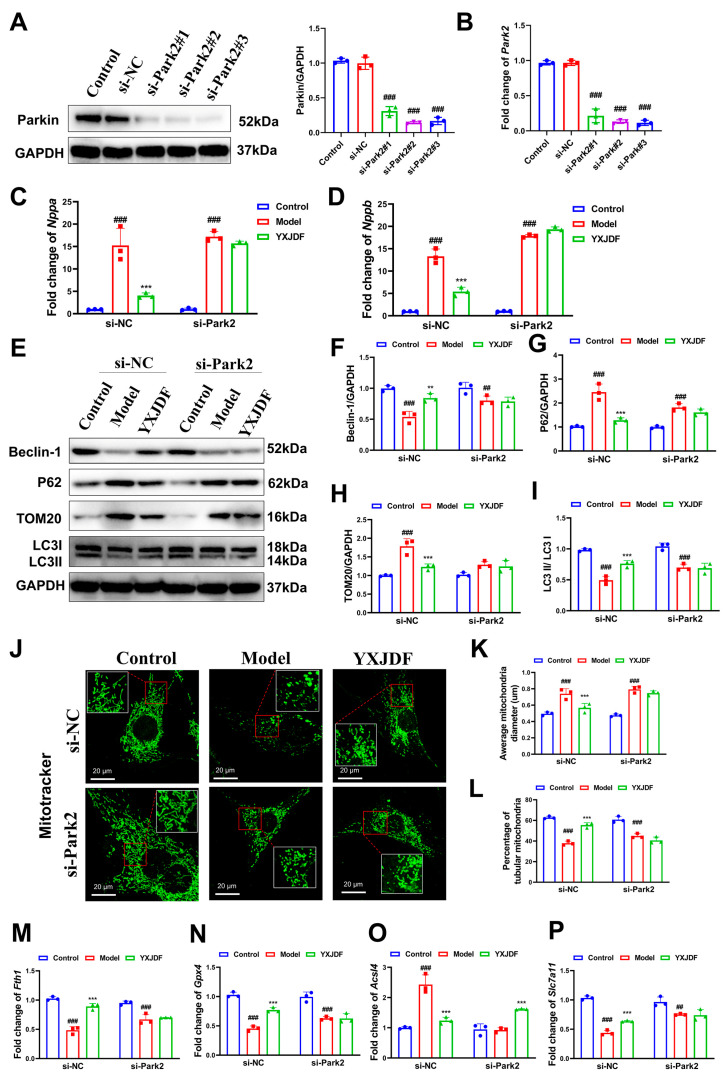
*Park2* knockdown weakens the protective effects of YXJDF on mitophagy and ferroptosis in HL-1 cardiomyocytes. (**A**,**B**) Quantitative real-time PCR (qRT-PCR) and Western blot analysis of *Park2* (the gene encoding Parkin) expression in HL-1 cardiomyocytes transfected with negative control siRNA (si-NC) or three independent siRNAs targeting *Park2* (si-Park2). si-Park2#2, which showed the highest knockdown efficiency at both mRNA and protein levels, was selected for subsequent experiments. (**C**,**D**) qRT-PCR analysis of the hypertrophic markers *Nppa* and *Nppb* in angiotensin II (Ang II)-stimulated HL-1 cardiomyocytes treated with YXJDF-containing serum under si-NC or si-Park2 conditions. (**E**) Representative Western blot bands of mitophagy-related proteins (Beclin-1, p62, translocase of outer mitochondrial membrane 20 (TOM20), and microtubule-associated protein 1 light chain 3 (LC3)-I and LC3-II). (**F**–**I**) Quantification of Beclin-1 (**F**), p62 (**G**), TOM20 (**H**), and LC3-II/LC3-I ratio (**I**) based on Western blot. (**J**) Representative MitoTracker staining images and (**K**,**L**) quantitative analysis of mitochondrial morphology, including average mitochondrial diameter and percentage of tubular mitochondria. (**M**–**P**) qRT-PCR analysis of ferroptosis-related genes, including *Fth1*, *Gpx4*, *Acsl4*, and *Slc7a11*. Data are expressed as mean ± SD (n = 3 biological replicates per group). ^##^ *p* < 0.01, ^###^ *p* < 0.001 vs. Control; ** *p* < 0.01, *** *p* < 0.001 vs. Model. Abbreviations: YXJDF, serum obtained from rats treated with QiShen granule; TMZ, trimetazidine; si-NC, small interfering RNA negative control; si-Park2, small interfering RNA targeting *Park2* (gene encoding Parkin).

To determine whether Parkin is functionally required for the protective effects of YXJDF rather than being merely associated with mitophagy activation, loss-of-function experiments were performed by silencing *Park2*, the gene encoding Parkin, in HL-1 cardiomyocytes. Three independent siRNAs targeting *Park2* were designed and tested, and si-Park2#2, which exhibited the highest knockdown efficiency at both the mRNA and protein levels, was selected for subsequent experiments (Figure 9A,B).

In cells transfected with control siRNA, YXJDF treatment markedly alleviated angiotensin II–induced cellular injury, as evidenced by significant reductions in the hypertrophic markers *Nppa* and *Nppb*. Notably, when *Park2* was silenced, the ability of YXJDF to suppress these hypertrophic responses was substantially weakened, indicating that the cardioprotective effects of YXJDF are compromised in the absence of Parkin (Figure 9C,D). At the level of mitochondrial quality control, YXJDF robustly enhanced mitophagy-related signaling in control cells, reflected by increased expression of Beclin-1 and the LC3-II/LC3-I ratio, along with reduced accumulation of p62 and TOM20. In contrast, these YXJDF-induced changes were markedly attenuated under *Park2* knockdown conditions, suggesting that the restoration of mitophagic activity by YXJDF requires functional Parkin signaling (Figure 9E–I). Consistently, MitoTracker staining demonstrated that the improvement of mitochondrial integrity and distribution elicited by YXJDF was evident in control cells but was significantly diminished when *Park2* expression was reduced (Figure 9J–L).

Given the close functional link between mitophagy and ferroptosis, we further examined whether *Park2* deficiency influences the anti-ferroptotic effects of YXJDF. In control cells, YXJDF effectively normalized the expression of ferroptosis-related regulators, including upregulation of *Fth1* and *Gpx4* and suppression of *Acsl4* and *Slc7a11* dysregulation. However, these protective effects were only partially retained in *Park2*-deficient cells, indicating that the ferroptosis-suppressing action of YXJDF is blunted when Parkin signaling is impaired (Figure 9M–P).

Collectively, these findings demonstrate that *Park2* knockdown reduces the efficacy of YXJDF in protecting cardiomyocytes against hypertrophy, mitochondrial dysfunction, and ferroptotic stress, supporting the conclusion that an intact PINK1/Parkin axis is required for the full cardioprotective effects of YXJDF.

### 2.8. LC-MS/MS Profiling and Molecular Docking Identify Potential Mitophagy-Modulating Components in YXJDF

**Figure 10:**
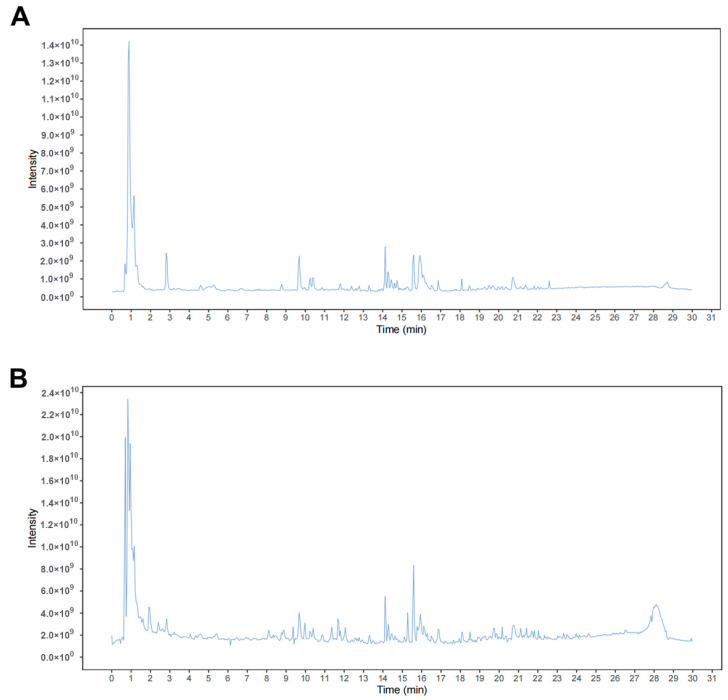
Liquid chromatography–tandem mass spectrometry (LC-MS/MS)-based identification of the chemical constituents in YXJDF. (**A**,**B**) Representative total ion chromatograms (TICs) of YXJDF extract in (**A**) negative and (**B**) positive ion modes obtained by LC-MS/MS analysis. Multiple distinct peaks indicate the presence of diverse bioactive compounds within the formulation. Abbreviations: YXJDF, Yixinjiedu formula.

Furthermore, LC-MS/MS profiling in both negative and positive ion modes was performed to characterize the chemical composition of YXJDF and to identify its major constituents. The total ion chromatograms revealed multiple distinct peaks, indicating the presence of numerous chemical compounds in the formulation (Figure 10A,B). Based on accurate mass measurements and fragmentation patterns, a total of 20 major compounds were identified, including Calycosin, Chlorogenic acid, Arglabin, Bisoxireno, Trifolirhizin, and Salvianolic acid A (Table 1).

**Figure 11:**
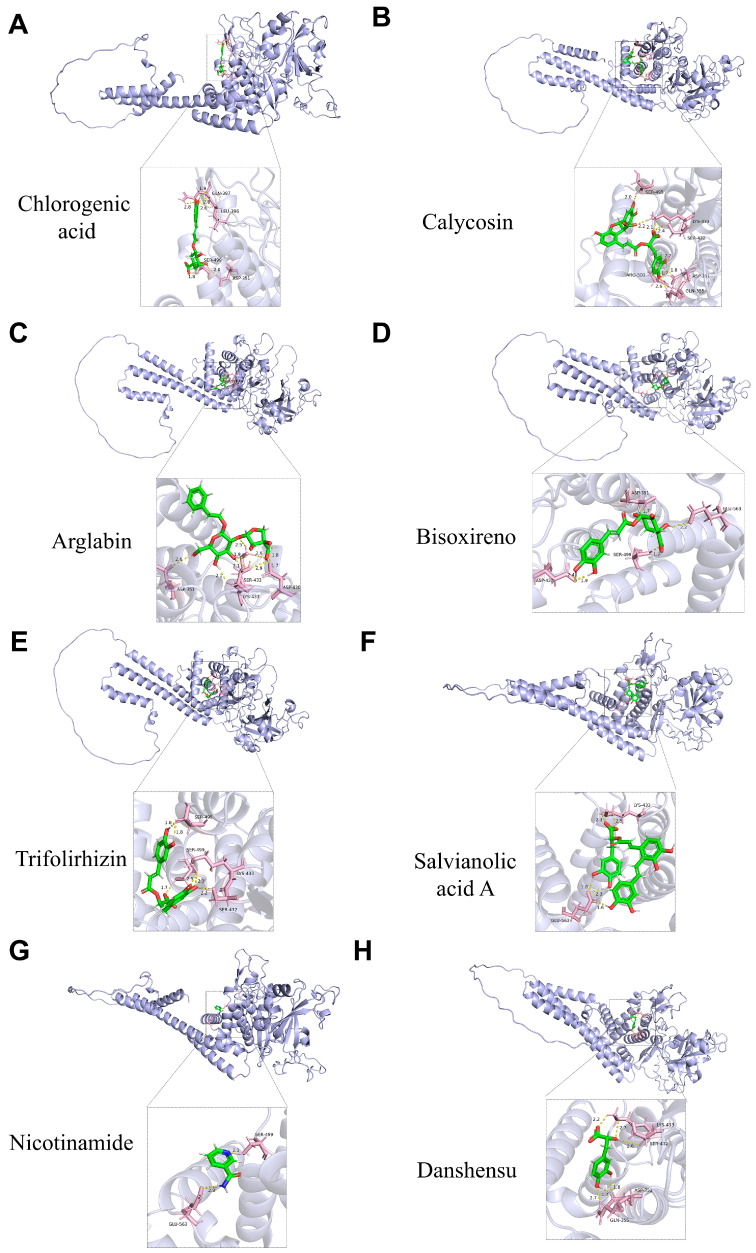
Molecular docking analysis of major active compounds in YXJDF with the core mitophagy regulator PTEN-induced putative kinase 1 (PINK1). (**A**–**H**) Predicted docking conformations of eight representative compounds with high binding affinity to PINK1. Binding pockets and key interaction residues are visualized. The compounds include: (**A**) Chlorogenic acid, (**B**) Calycosin, (**C**) Arglabin, (**D**) Bisoxireno, (**E**) Trifolirhizin, (**F**) Salvianolic acid A, (**G**) Nicotinamide, and (**H**) Danshensu.

To explore potential interactions between these compounds and the predicted core target PINK1, molecular docking analysis was conducted using AutoDock Vina. The binding affinity values (kcal/mol) revealed strong interactions between PINK1 and several constituents. Notably, Chlorogenic acid (−9.5987 kcal/mol), Calycosin (−8.9726 kcal/mol), Arglabin (−8.9721 kcal/mol), and Bisoxireno (−8.6026 kcal/mol) exhibited the highest binding affinities (Table 2), suggesting their potential role in regulating mitophagy through PINK1. Structural visualization of the docking complexes confirmed stable binding conformations within the active site of PINK1 (Figure 11A–H). These findings validate that multiple compounds in YXJDF possess the structural capability to interact with PINK1, thereby providing a molecular basis for the observed activation of the PINK1/Parkin-mediated mitophagy pathway in pressure overload-induced HF.

## 3. Discussion

As a major global health burden, HF can result from a range of cardiovascular disorders, including acute myocardial infarction, persistent hypertension, valvular dysfunction, and prolonged hemodynamic stress [22]. Among these, pressure overload is a key contributor to HF with preserved or reduced ejection fraction, which is frequently associated with progressive ventricular hypertrophy, maladaptive remodeling, and contractile dysfunction [23]. YXJDF has shown clinical efficacy in the treatment of chronic HF [19,24]. However, mechanistic studies of YXJDF have largely focused on ischemia-related models [25,26]. Accumulating evidence has demonstrated that YXJDF or its standardized preparation, QSG, exerts broad cardioprotective effects across multiple cardiovascular disease contexts. Previous studies have shown that QSG alleviates doxorubicin-induced cardiotoxicity by suppressing ferroptosis via activation of the NRF2 signaling pathway, highlighting its role in regulating oxidative stress-driven cell death [27]. In addition, QSG has been reported to protect against myocardial infarction–induced injury by inhibiting NLRP3 inflammasome activation and pyroptosis, indicating its capacity to modulate inflammatory forms of regulated cell death [20]. Other investigations further revealed that QSG preserves mitochondrial function and redox homeostasis by regulating Sirtuin3 [28], and improves cardiac function in HF models by modulating intestinal microecology and systemic metabolism [21]. However, its role in pressure overload-induced HF and the molecular mechanisms involved remain insufficiently understood. In particular, whether YXJDF modulates cardiomyocyte death pathways such as ferroptosis or regulates mitochondrial quality control has not been systematically investigated. In this study, using both in vivo (TAC-induced murine HF model) and in vitro (Ang II-stimulated HL-1 cardiomyocytes) systems, we showed that YXJDF significantly improved cardiac function, reduced oxidative stress, suppressed ferroptosis, and enhanced mitochondrial quality control. These findings were further supported by network pharmacology analysis, which identified PINK1 as a key regulatory target. LC-MS/MS and molecular docking results further identified several YXJDF compounds with high binding affinity to PINK1.

TAC is a widely established and reproducible model for inducing pressure overload in rodents, effectively mimicking the pathophysiological progression of human HF, including cardiac hypertrophy, fibrosis, and systolic dysfunction [29,30]. Previous studies have utilized the TAC model to investigate molecular mechanisms underlying mechanical stress-induced cardiac remodeling and to evaluate the efficacy of pharmacologic interventions [31,32]. In our present study, TAC surgery successfully induced typical HF phenotypes, as evidenced by reduced ejection fraction, increased cardiac mass index, and prominent histopathological abnormalities. YXJDF treatment markedly improved these parameters, suggesting that it confers protective effects in the context of chronic pressure overload.

Pressure overload imposes sustained mechanical stress on cardiomyocytes, which triggers a cascade of maladaptive responses, including hypertrophic signaling, mitochondrial dysfunction, and elevated ROS production. Excessive ROS not only causes oxidative damage to lipids, proteins, and nucleic acids but also contributes to the induction of ferroptosis [33]. Ferroptosis is a regulated form of iron-dependent cell death, which is characterized by lipid peroxidation and mitochondrial shrinkage [34]. Recent studies have highlighted the central role of ferroptosis in the progression of HF, where impaired antioxidant capacity and disrupted iron homeostasis exacerbate cardiomyocyte loss and contractile dysfunction [35]. In this study, pressure overload resulted in marked ROS accumulation, elevated MDA levels, and reduced antioxidant capacity, accompanied by dysregulation of ferroptosis-related regulators. Specifically, ACSL4, which promotes incorporation of polyunsaturated fatty acids into membrane phospholipids and enhances susceptibility to lipid peroxidation [36], was upregulated, whereas FTH1, a key iron-storage protein that limits redox-active iron availability [37], was downregulated. These molecular alterations indicate a myocardial environment prone to ferroptosis. Importantly, the suppression of ferroptosis by YXJDF was further supported by C11-BODIPY–based lipid peroxidation assays, which provide a ferroptosis-specific functional readout and closely mirrored the changes observed in ACSL4 expression. Notably, YXJDF treatment reversed these alterations, suggesting that it suppresses ferroptosis by simultaneously reducing lipid peroxidation susceptibility and restoring iron homeostasis, thereby limiting ferroptotic cardiomyocyte death.

To further elucidate the molecular mechanisms underlying the cardioprotective effects of YXJDF, a network pharmacology approach was employed to systematically predict its potential targets and key signaling pathways. Network pharmacology has emerged as a powerful tool for deciphering the multi-component, multi-target, and multi-pathway characteristics of TCM formulations [38]. In recent years, it has been widely used to investigate the pharmacological basis of herbal prescriptions in cardiovascular and metabolic diseases. For instance, Zhao et al. applied network pharmacology to reveal that cardioprotective effects of SanQi-DanShen may involve the inhibition of the PI3K/AKT signaling pathway in coronary heart disease [39]. More recently, Yang et al. demonstrated that the classical formula Shenmai injection alleviates post-myocardial infarction by targeting the PPARα/SIRT1/PGC1α pathway, as identified through network pharmacology [40]. Among the predicted targets of our network pharmacology analysis, PINK1 was identified as a candidate of particular interest. Although it was not ranked as the top hub gene by degree centrality in the PPI network, PINK1 emerged as a significantly enriched target in GO and KEGG analyses, particularly in pathways related to mitophagy and oxidative stress regulation. Given its established role in maintaining mitochondrial homeostasis and removing damaged mitochondria via the PINK1/Parkin axis, we prioritized PINK1 for further validation.

PINK1 functions as a key sensor of mitochondrial depolarization and orchestrates mitophagy by recruiting and activating the E3 ubiquitin ligase Parkin to impaired mitochondria [41]. This PINK1/Parkin-dependent pathway is essential for the selective removal of dysfunctional mitochondria and has been implicated in the pathogenesis of cardiac hypertrophy and HF [42]. In addition, efficient mitophagic flux requires coordinated autophagosome formation, in which ATG16L plays a critical role by facilitating LC3 lipidation and autophagosome elongation [43]. In our study, pressure overload and Ang II stimulation led to downregulation of PINK1, Parkin, and ATG16L, indicating impaired mitophagy and defective mitochondrial turnover rather than simple alterations in autophagy initiation. YXJDF treatment restored the expression of these key regulators at both the transcriptional and protein levels in vivo and in vitro, supporting the conclusion that YXJDF enhances functional mitophagy and improves mitochondrial quality control. More importantly, autophagic flux analyses using BafA1 further confirmed that the observed changes in LC3, p62, and mitochondrial markers reflected enhanced mitophagic flux rather than impaired autophagosome degradation.

Importantly, accumulating evidence suggests that ferroptosis and mitophagy are mechanistically interconnected rather than independent processes [44,45]. Impaired mitophagy leads to the accumulation of damaged mitochondria, excessive mitochondrial ROS production, and lipid peroxidation, thereby creating a permissive environment for ferroptosis. Conversely, ferroptotic damage further exacerbates mitochondrial dysfunction, forming a deleterious feed-forward loop under conditions of chronic pressure overload [46]. Our findings suggest that YXJDF suppresses ferroptosis, at least in part, through restoration of mitophagy via the PINK1/Parkin axis. Consistently, genetic silencing of Parkin markedly attenuated the protective effects of YXJDF on cardiomyocyte hypertrophy, mitophagy restoration, and ferroptosis suppression, indicating that Parkin is a necessary mediator rather than a secondary downstream marker in this process. By enhancing mitochondrial clearance and reducing oxidative stress, YXJDF indirectly limits ferroptotic signaling, providing an integrated mechanistic explanation for its cardioprotective effects.

Consistent with these mechanistic insights, LC-MS/MS analysis identified multiple bioactive compounds within YXJDF, and molecular docking further revealed that several compounds, including chlorogenic acid, calycosin, arglabin, and bisoxireno, which exhibited strong binding affinities with PINK1, supporting their potential roles in modulating PINK1/Parkin signaling. These findings reinforce the multi-component, multi-target nature of TCM and suggest that coordinated modulation of mitochondrial quality control may underlie its therapeutic efficacy in complex cardiovascular disorders such as HF.

Despite these encouraging findings, several limitations should be acknowledged. First, the present study was conducted using a specific commercially available and standardized formulation of the Yixinjiedu formula (QSG); variations in herbal composition and quality control among products from different manufacturers may exist, and thus the observed effects should be interpreted within the context of the studied preparation. Second, our mechanistic focus centered on the PINK1/Parkin axis; whether YXJDF regulates mitophagy through additional pathways (e.g., BNIP3, FUNDC1) remains unexplored. Third, while LC-MS/MS and docking analyses suggested candidate active compounds, further experimental validation (e.g., individual compound intervention, target engagement assays) is warranted. Finally, our study did not assess long-term safety or systemic metabolic effects of YXJDF.

## 4. Materials and Methods

### 4.1. Drug Preparation

YXJDF, commercially available as QiShen Granule (QSG), consists of five traditional Chinese medicinal herbs: *Astragalus membranaceus*, *Salvia miltiorrhiza*, *Paeoniae rubra*, *Ligusticum chuanxiong*, and *Glycyrrhiza uralensis*. QSG was purchased from Beijing Tongrentang Co., Ltd. (Beijing, China), an officially certified pharmaceutical manufacturer. The product is approved by the National Medical Products Administration of China and manufactured in accordance with the standards of the Chinese Pharmacopoeia [47]. All herbal components have been taxonomically authenticated and quality-controlled by the manufacturer. All experiments were performed using the same production batch to ensure consistency. For animal experiments, QSG was freshly prepared each day by dissolving the required amount in distilled water to obtain suspensions suitable for oral gavage. Trimetazidine (TMZ; H20213217), used as a reference drug to provide mechanistic comparison, was purchased from the First Affiliated Hospital of Guangzhou University of Chinese Medicine.

### 4.2. Animal Model and Treatment

Male C57BL/6 mice (8 weeks old, 20–25 g) obtained from Guangdong Medical Laboratory Animal Center (Guangzhou, China) were housed under specific pathogen-free (SPF) conditions (temperature: 23 ± 2 °C; humidity: 55 ± 5%; 12 h light/dark cycle) with free access to food and water. All mice received a 1-week acclimatization period before experiments. Mice were randomly assigned to six groups (n = 6 per group): Sham, TAC, YXJDF-L, YXJDF-M, YXJDF-H, and TMZ.

Pressure overload-induced heart failure was established via transverse aortic constriction (TAC). In brief, mice were anesthetized with an intraperitoneal injection of pentobarbital sodium (50 mg/kg). After confirming loss of pedal reflex, hair over the neck and chest was shaved and disinfected with 75% ethanol. Mice were fixed in a supine position using adhesive tape, and a median sternotomy was performed to expose the transverse aorta. A 27-gauge needle was placed adjacent to the aorta, and the vessel was ligated three times using a 6-0 silk suture. The needle was then gently removed to achieve constriction. Sham mice underwent the same procedure without ligation. The study was approved by the Animal Care and Use Committee of Guangzhou University of Chinese Medicine (Approval No. 20240307001). No animals were excluded from the study after group allocation. All mice that successfully underwent the surgical procedure and survived until the predefined experimental endpoints were included in the subsequent analyses. Exclusion criteria were established a priori and included unexpected death related to surgical complications, severe postoperative infection, or technical failure during TAC surgery. No unexpected adverse events occurred during the study. Mild and transient postoperative stress responses were observed following TAC surgery, which resolved during the recovery period and did not require additional intervention. No data points were excluded based on outcome measures.

Postoperative care included daily intraperitoneal injections of penicillin sodium (18,000 U/kg) for 3 days to prevent infection. Starting from the second day after surgery, mice in the YXJDF-L, YXJDF-M, and YXJDF-H groups were administered low (2.83 g/kg/day), medium (5.66 g/kg/day), and high (11.3 g/kg/day) doses of QSG, respectively, via oral gavage. The selected dose range was based on previously published studies demonstrating significant cardioprotective effects and safety in experimental cardiac injury models [25,48]. Mice in the TMZ group received TMZ at 20 mg/kg/day by oral gavage, dissolved in distilled water. TMZ at 20 mg/kg/day was used as a positive control. The selected dose was based on previous experimental studies reporting cardioprotective and metabolic regulatory effects in murine models of cardiac injury [49]. Mice in the Sham and TAC groups received an equal volume of distilled water. Drug or vehicle administration was performed once daily for 8 consecutive weeks. Due to the nature of the experimental procedures, blinding was not applied during outcome assessment. However, all analyses were performed using predefined criteria and standardized protocols to minimize subjective bias.

The sample size was determined based on previous studies using the TAC mouse model to evaluate cardiac dysfunction and pharmacological interventions, in which group sizes of 5–8 animals were sufficient to detect biologically meaningful differences in cardiac function and molecular endpoints. Considering ethical principles to minimize animal use and practical feasibility, six mice per group were selected, which was deemed adequate to ensure reproducibility and statistical reliability for the primary outcome measures. All were included in functional, histological, biochemical, and molecular analyses unless otherwise specified. No formal a priori statistical power calculation was performed.

### 4.3. Echocardiographic Analysis

Cardiac function was assessed using a high-frequency ultrasound imaging system (Vevo 2100, VisualSonics Inc., Toronto, ON, Canada) with a 30 MHz linear transducer. Left ventricular function and structure were evaluated by measuring the left ventricular ejection fraction (LVEF), fractional shortening (LVFS), end-diastolic and end-systolic internal dimensions (LVIDd and LVIDs), end-diastolic and end-systolic volumes (LVEDV and LVESV), and left ventricular mass (LV mass). Each parameter was calculated as the average of at least three consecutive cardiac cycles. All analyses were performed by an investigator blinded to the experimental groups using Vevo LAB software (version 5.7.0).

### 4.4. Tissue Collection and Processing

Following echocardiographic examination, mice were euthanized by cervical dislocation under deep anesthesia induced by intraperitoneal pentobarbital sodium (50 mg/kg). The thoracic cavity was immediately opened, and the heart was carefully excised, rinsed in cold phosphate-buffered saline (PBS), and blotted dry. Total heart weight (HW) was recorded using an analytical balance. The ratio of heart weight to body weight (HW/BW) was calculated to evaluate cardiac hypertrophy. After weighing, the atria and right ventricle were removed, and the left ventricle was isolated. The left ventricular tissue was then divided into two portions: one portion was snap-frozen in liquid nitrogen and stored at −80 °C for biochemical and molecular analyses, while the remaining portion was fixed in 4% paraformaldehyde overnight at 4 °C, followed by dehydration, paraffin embedding, and sectioning into 4-μm-thick slices for subsequent histological analysis.

### 4.5. Histological Staining and Analysis

Hematoxylin and eosin (H&E) staining was performed using a standard protocol. Sections were deparaffinized, rehydrated, and stained with hematoxylin for nuclear visualization, followed by eosin counterstaining. Histological changes, including ventricular chamber enlargement, myocardial fiber disarray, and inflammatory cell infiltration, were observed under a light microscope (Olympus BX53, Tokyo, Japan)

Wheat germ agglutinin (WGA) staining was carried out to assess cardiomyocyte cross-sectional area (CSA). After deparaffinization, antigen retrieval was performed by microwaving sections in EDTA buffer (pH 8.0). Sections were cooled to room temperature and encircled with a Dako pen. WGA staining solution (I3310, Beijing Solarbio, Beijing, China) was applied to the tissue and incubated at 37 °C for 30 min in the dark. After three washes with PBST, sections were mounted using anti-fade mounting medium and imaged using an Olympus IX73 fluorescence microscope (Olympus, Tokyo, Japan). ImageJ software (version 1.41) was used to quantify CSA by tracing WGA-positive membrane boundaries. Analysis was performed in six randomly selected fields per section. All image analyses were performed by two independent observers blinded to group allocation.

Masson’s trichrome staining was carried out using a commercial kit (G1006, Servicebio, Wuhan, China) according to the manufacturer’s protocol. Paraffin sections were dewaxed, rehydrated, and stained with Weigert’s iron hematoxylin for 8 min to visualize nuclei. After rinsing, sections were differentiated in acidic ethanol for 15 s and washed in water. Masson blue solution was applied for 5 min, followed by rinsing and incubation with ponceau-acid fuchsin solution for 5 min to stain muscle fibers. Sections were then differentiated in phosphomolybdic acid solution and counterstained with aniline blue for collagen visualization. A final wash in 1% acetic acid was performed before dehydration with graded ethanol, clearing, and mounting. Collagen fibers appeared blue and muscle fibers red. Fibrotic areas were quantified using ImageJ software by calculating the percentage of collagen-stained area relative to the total myocardial area from six randomly selected fields per section. Images were acquired under a bright-field microscope (Olympus IX73, Tokyo, Japan), and all analyses were performed by investigators blinded to group assignments.

### 4.6. Preparation of YXJDF-Containing Serum

Male Sprague-Dawley rats were randomly divided into a control group and a YXJDF-treated group (n = 6 per group). The YXJDF group received oral administration of QSG suspension at a dose of 7.82 g/kg/day for five consecutive days, while the control group received an equal volume of sterile water. Two hours after the final administration, rats were anesthetized, and blood was collected from the abdominal aorta. Whole blood was incubated at 4 °C for 2 h and centrifuged at 15,000 rpm for 5 min to isolate serum. The collected serum was heat-inactivated at 56 °C for 1 h in a water bath, followed by filtration through a 0.22 μm filter. The inactivated serum was then aliquoted and stored at −20 °C for later use in cell culture experiments.

The dose of QSG used for serum preparation (7.82 g/kg/day) was calculated based on body surface area normalization from the high-dose mouse regimen (11.3 g/kg/day), using standard interspecies conversion coefficients (mouse: 9.1; rat: 6.3).

### 4.7. Cell Culture and In Vitro Injury Model

HL-1 cardiomyocytes (Procell, Wuhan, China) were cultured in Minimum Essential Medium (MEM; Gibco, Waltham, MA, USA) supplemented with 10% fetal bovine serum (FBS; Gibco) and 1% penicillin-streptomycin solution (Gibco). Cells were maintained at 37 °C in a humidified atmosphere containing 5% CO_2_. Upon reaching approximately 80% confluence, cells were seeded into culture plates for further experiments.

Cells were divided into four groups: control, model, YXJDF, and TMZ groups. Ang II, YXJDF, and TMZ groups were treated with 150 nM Ang II (Sigma-Aldrich, St. Louis, MO, USA) for 24 h to induce cellular injury. The YXJDF group received 10% YXJDF-containing serum in MEM, while the control and Ang II groups received MEM supplemented with 10% normal rat serum.

### 4.8. Autophagic Flux Analysis

Autophagic flux analysis was performed to distinguish enhanced mitophagy from impaired lysosomal degradation. HL-1 cardiomyocytes were treated with angiotensin II and YXJDF-containing serum as described above, and bafilomycin A1 (BafA1; Sigma-Aldrich) was applied at a final concentration of 100 nM for the last 4 h before cell collection to inhibit lysosomal acidification and autophagosome–lysosome fusion. Cells were then harvested for Western blot analysis of mitophagy- and autophagy-related markers.

### 4.9. Phalloidin Staining

Cell morphology and cytoskeletal structure were analyzed by phalloidin staining. HL-1 cardiomyocytes were seeded onto sterilized glass coverslips and treated according to experimental groupings. After treatment, cells were washed twice and then fixed in 4% paraformaldehyde for 20 min at room temperature. After being washed again with PBS, cells were permeabilized with 0.1% Triton X-100 for 10 min and blocked with 1% bovine serum albumin (BSA) for 30 min. Next, cytoskeletal F-actin was stained with Alexa Fluor™ 488-conjugated phalloidin (Invitrogen, A12379, Carlsbad, CA, USA) at a concentration of 5 μg/mL for 1 h at 37 °C in the dark. Nuclei were counterstained with DAPI. Finally, coverslips were mounted after removing excess liquid and visualized using a laser scanning confocal microscope (Leica, Düsseldorf, Germany).

### 4.10. MitoTracker STAINING

To assess mitochondrial morphology and membrane potential in HL-1 cardiomyocytes, MitoTracker™ Green solution (Beyotime, C1048, Shanghai, China) was used according to the manufacturer’s instructions. Cells were incubated with 200 nM MitoTracker working solution at 37 °C for 30 min in the dark. After staining, cells were washed three times with prewarmed PBS and fixed with 4% paraformaldehyde for 10 min at room temperature. Fluorescent images were captured using a confocal laser scanning microscope (Leica).

### 4.11. C11-BODIPY Staining

Lipid peroxidation was assessed using the ferroptosis-sensitive fluorescent probe C11-BODIPY 581/591 (Invitrogen, D3861). HL-1 cardiomyocytes were seeded on glass coverslips and treated according to the indicated experimental conditions. After treatment, cells were incubated with C11-BODIPY working solution (2 μM) in serum-free medium at 37 °C for 30 min in the dark. Subsequently, cells were washed three times with prewarmed PBS to remove excess dye. Fluorescent images were acquired using a confocal laser scanning microscope (Leica) with identical acquisition settings across groups.

Oxidation of C11-BODIPY was evaluated by measuring the shift from red fluorescence (reduced form) to green fluorescence (oxidized form). Quantitative analysis was performed using ImageJ software by calculating the ratio of green to red fluorescence intensity for each field, which reflects the extent of lipid peroxidation.

### 4.12. Bioinformatics Analysis

The active components of YXJDF were retrieved from the TCMSP database (https://tcmsp.91medicine.cn/TCMSP) based on the criteria of oral bioavailability (OB ≥ 30%) and drug-likeness (DL ≥ 0.18), and further supplemented by manual curation from the HERB database (http://herb.ac.cn/). All collected components were mapped to their potential targets using the UniProt database (https://www.uniprot.org/) with species restricted to “Homo sapiens”. Heart failure-related genes were identified using the GeneCards (https://www.genecards.org/) and OMIM (https://omim.org/) databases with “heart failure” as the keyword. Genes with a GeneCards relevance score ≥ 10 were selected. Overlapping genes between YXJDF-related targets and heart failure-associated genes were defined as potential therapeutic targets. Next, the component-target-disease interaction network was constructed using Cytoscape 3.9.1. Protein–protein interaction (PPI) networks of the overlapping genes were generated using the Search Tool for the Retrieval of Interacting Genes/Proteins (STRING) database (https://string-db.org/) with a confidence score > 0.7.

Gene Ontology (GO) and Kyoto Encyclopedia of Genes and Genomes (KEGG) pathway enrichment analyses were performed using the Metascape platform (https://metascape.org/) with *p* < 0.05 as the cutoff. Bubble charts and enrichment plots were generated using the bioinformatics visualization platform (http://www.bioinformatics.com.cn/).

### 4.13. Western Blot Analysis

Total protein was extracted from mouse heart tissues and HL-1 cells using RIPA lysis buffer (RG235174, Thermo, Waltham, MA, USA) supplemented with protease and phosphatase inhibitors (25× cocktail, RE2173411, Thermo), followed by determining protein concentrations with a BCA Protein Assay Kit (RJ240544, Thermo). Next, equal amounts of denatured protein were loaded onto 8–15% SDS-PAGE gels and electrophoresed under constant voltage, then transferred to methanol-activated PVDF membranes (Millipore, Burlington, MA, USA) via wet transfer at 100 V for 70–90 min under ice-cold conditions. Then, membranes were blocked with 5% non-fat milk in TBST for 1 h at room temperature and incubated overnight at 4 °C with primary antibodies. After three washes in TBST, membranes were incubated with HRP-conjugated secondary antibodies for 1 h at room temperature. Protein bands were visualized using enhanced chemiluminescence reagents (Millipore) and captured with a Bio-Rad ChemiDoc imaging system, Bio-Rad, Hercules, CA, USA. Band intensities were quantified using ImageJ software and normalized to GAPDH. Detailed information on all primary and secondary antibodies used in this study, including sources, catalog numbers, and dilutions, is provided in Supplementary Table S1.

### 4.14. Oxidative Stress Analysis

Oxidative stress levels in cardiac tissues were evaluated using commercially available assay kits according to the manufacturers’ instructions. Left ventricular tissues were homogenized in ice-cold buffer, and the supernatants were collected for analysis after centrifugation. The levels of malondialdehyde (MDA), reduced glutathione (GSH), and total superoxide dismutase (T-SOD) activity were measured using assay kits purchased from Nanjing Jiancheng Bioengineering Institute (Nanjing, China). Absorbance was measured using a microplate reader, and all values were normalized to the total protein concentration determined by the BCA method.

### 4.15. RNA Extraction and Quantitative Real-Time PCR (qRT-PCR) Analysis

Total RNA was extracted from mouse heart tissues and HL-1 cells using Trizol reagent (Invitrogen) following the manufacturer’s instructions, and RNA concentration was measured using a NanoDrop spectrophotometer (Thermo Fisher Scientific, Waltham, MA, USA). After synthesizing cDNA using a 5× PrimeScript™ RT Master Mix Kit (Takara, Kusatsu, Japan), qPCR was carried out using TB Green Premix Ex Taq II (Takara) with the following thermal cycling conditions: initial denaturation at 95 °C for 30 s, followed by 40 cycles of 95 °C for 5 s, 58–60 °C for 30 s, and 72 °C for 45 s. Melt curve analysis was performed to confirm specificity. Relative mRNA expression was calculated using the 2^−ΔΔCt^ method [50] and normalized to β-actin. Primer sequences are listed in Supplementary Table S2.

### 4.16. Liquid Chromatography–Mass Spectrometry (LC-MS/MS) Analysis

The chemical profile of the YXJDF formula was characterized using LC-MS/MS. The YXJDF extract was prepared by dissolving 0.3 g of QSG powder from a single production batch in 8 mL of 50% methanol aqueous solution. The mixture was sonicated at 45 °C for 30 min, allowed to stand for 5 min, and then centrifuged at 1000 rpm for 7 min. The resulting supernatant was filtered through a 0.22 μm microporous membrane and transferred into a 2.0 mL autosampler vial.

Chromatographic separation was performed on a Waters BEH C18 column (100 mm × 2.1 mm, 1.7 μm) (Waters Corporation, Milford, MA, USA) using gradient elution. The mobile phase consisted of solvent A (0.1% formic acid in acetonitrile) and solvent B (water). The gradient program ranged from 5% to 95% solvent A over 60 min at a flow rate of 0.2 mL/min. The injection volume was 5 μL. Mass spectrometry was performed using an electrospray ionization (ESI) source operated in both positive and negative ion modes. The scan range was set to *m*/*z* 50–2000. Major constituents were confidently identified based on retention time and mass spectra, supported by compound information from TCMSP and HERB databases. LC-MS/MS profiling was used as a qualitative chemical fingerprinting approach to support component identification rather than for quantitative standardization.

### 4.17. Molecular Docking Analysis

Molecular docking analysis was conducted to evaluate the binding affinity between key active components of YXJDF and the core target PINK1. Three-dimensional structures of the ligands were downloaded from the PubChem database and converted to PDB format using Open Babel. The crystal structure of human PINK1 was obtained from the RCSB PDB database and preprocessed by removing water molecules and adding hydrogen atoms. Docking simulations were performed using AutoDock Vina (version 1.1.2). The search grid was defined around the active pocket of PINK1, and docking scores were calculated as binding free energy (kcal/mol). Visualization of binding conformations was performed using PyMOL software (version 3.1).

### 4.18. Statistical Analysis

All experimental data were expressed as mean ± standard deviation (SD). Statistical analyses were performed using GraphPad Prism 9.0 (GraphPad Software, San Diego, CA, USA). Unless otherwise specified, “n” represents the number of independent biological replicates, defined as individual animals for in vivo experiments or independently treated cell cultures for in vitro experiments. Technical replicates were averaged prior to statistical analysis.

The primary endpoints of this study were predefined as cardiac functional parameters assessed by echocardiography and core mechanistic readouts related to mitophagy and ferroptosis. Secondary endpoints included histological, biochemical, and exploratory molecular analyses that were used to support mechanistic interpretation.

Data distribution and variance homogeneity were assessed prior to statistical analysis. Normality is assumed for the applied parametric tests based on experimental design and inspection of data distribution. In cases where data did not meet the assumptions of normality or homogeneity of variance, appropriate non-parametric tests were considered. No data transformation was applied. Comparisons between two groups were conducted using the unpaired two-tailed Student’s *t*-test. For comparisons among three or more groups, one-way analysis of variance (ANOVA) followed by Bonferroni’s post hoc test was applied.

No formal a priori power calculation was performed. Sample sizes were determined based on previous studies using similar experimental models, which demonstrated sufficient sensitivity to detect biologically meaningful differences while minimizing animal use. Given the hypothesis-driven nature of the study, statistical comparisons were limited to predefined contrasts, and secondary endpoints were interpreted in a supportive rather than confirmatory manner to reduce the risk of false-positive findings. A *p*-value < 0.05 was considered statistically significant.

## 5. Conclusions

In conclusion, our study demonstrated for the first time that YXJDF exerts robust cardioprotective effects against pressure overload-induced HF by concurrently alleviating ferroptosis and restoring PINK1/Parkin-mediated mitophagy. These results not only provide new mechanistic insights into the cardioprotective effects of YXJDF but also highlight the therapeutic potential of targeting ferroptosis-mitophagy crosstalk in HF.

## Figures and Tables

**Table 1:** The chemical ingredients of YXJDF have been identified through formulation analysis using LC-MS/MS.

Number	Name	Formula	Median *m*/*z*	Median RT
1	Calycosin	C_16_H_12_O_5_	283.06	1012.39
2	Salvianolic acid A	C_26_H_22_O_10_	493.12	967.94
3	Danshensu	C_9_H_10_O_5_	197.05	171.42
4	Rosmarinic acid	C_18_H_16_O_8_	359.08	936.56
5	Lithospermic acid	C_27_H_22_O_12_	537.10	858.57
6	N-Acetyl-L-glutamic acid	C_7_H_11_NO_5_	188.06	71.49
7	Formononetin	C_16_H_12_O_4_	267.07	1108.90
8	Citric acid	C_6_H_8_O_7_	191.02	68.946
9	Loganic acid	C_16_H_24_O_10_	375.13	625.54
10	Protocatechualdehyde	C_7_H_6_O_3_	137.02	314.48
11	(1r,3R,4s,5S)-4-{[(2E)-3-(3,4-Dihydroxyphenyl)-2-propenoyl]oxy}-1,3,5-trihydroxycyclohexanecarboxylic acid	C_16_H_18_O_9_	377.08	580.21
12	Arglabin	C_15_H_18_O_3_	247.13	1123.33
13	Bisoxireno[4,5:8,9]cyclodeca[1,2-b]furan, 1a,2,6,6a,7a,8,9,9a-octahydro-1a,5,7a-trimethyl-	C_15_H_20_O_3_	231.14	1204.20
14	Kahweol	C_20_H_26_O_3_	315.19	1327.23
15	trifolirhizin	C_22_H_22_O_10_	469.11	1110.09
16	Nicotinamide	C_6_H_6_N_2_O	123.06	65.13
17	5-Hydroxymethylfurfural	C_6_H_6_O_3_	127.04	144.26
18	Chlorogenic acid	C_16_H_18_O_9_	355.10	613.98
19	Bullatine G	C_22_H_31_NO_3_	358.24	487.01
20	(2R,3S,4S,5R,6R)-5-[(2S,3R,4R)-3,4-dihydroxy-4-(hydroxymethyl)oxolan-2-yl]oxy-2-(hydroxymethyl)-6-(2-phenylethoxy)oxane-3,4-diol	C_19_H_28_O_10_	417.17	862.43

**Table 2:** Molecular docking between key active ingredients of YXJDF and PINK1.

Molecule	Affinity (kcal/mol)
Chlorogenic acid	−9.5987
Calycosin	−8.97261
Arglabin	−8.97219
Bisoxireno	−8.60261
Trifolirhizin	−7.72278
Salvianolic acid A	−7.6879
N-Acetyl-L-glutamic acid	−7.11161
5-Hydroxymethylfurfural	−7.08286
Nicotinamide	−6.77608
Kahweol	−6.58219
Citric acid	−6.38492
Bullatine G	−6.37235
Formononetin	−6.26974
Rosmarinic acid	−5.90823
Danshensu	−5.3848
Lithospermic acid	−4.591494
Protocatechualdehyde	−4.471596

## Data Availability

The data used to support the findings of this study are available from the corresponding author upon request.

## Supplementary Materials

The following supporting information can be downloaded at: https://www.mdpi.com/article/10.3390/ph19030360/s1, Figure S1: YXJDF promotes mitochondrial turnover through an increase in mitophagic flux; Table S1: The antibodies used in the study; Table S2: Primer sequences.
