# Supplementary material for: Yixinjiedu Formula Attenuates Pressure Overload-Induced Cardiac Dysfunction by Suppressing Ferroptosis and Restoring Mitophagy via the PINK1/Parkin Axis
Source: Pharmaceuticals (Basel). 2026 Feb 25;19(3):360. doi: 10.3390/ph19030360 (PMC13028673; doi:10.3390/ph19030360)
Supplement: Supplementary file 1 [file pharmaceuticals-19-00360-s001.zip › pharmaceuticals-4075594-tables S1 and S2.pdf]

Supplementary Table S1 The antibodies used in this research

Antibody	Manufacturer	Cat.no
Collagen I	Proteintech	14695-1-AP
Collagen III	Proteintech	22734-1-AP
Fibronectin	Proteintech	15613-1-AP
α-SMA	Proteintech	14395-1-AP
FTH1	Proteintech	10727-1-AP
COX2	Proteintech	12375-1-AP
NOX1	Proteintech	17772-1-AP
GPX4	Proteintech	14432-1-AP
NRF2	Proteintech	16396-1-AP
SLC7A11	Proteintech	26864-1-AP
ACSL4	Proteintech	22401-1-AP
PINK1	Proteintech	23274-1-AP
Parkin	Proteintech	66674-1-lg
Beclin-1	Proteintech	11306-1-AP
ATG16L	Proteintech	67943-1-Ig
LC3	Proteintech	14600-1-AP
GAPDH	Cell Signaling Technology	2118S

TOM20	Abcam	ab283317
P62	Abcam	Ab56416

Supplementary Table S2 Primer sequences

**Gene**	**Sequence**	
ANP	Forward	GCTTCGGGGGTAGGATTGAC
	Reverse	CACACCACAAGGGCTTAGGA
BNP	Forward	CGGATCCGTCAGTCGTTTGG
	Reverse	AAAGAGACCCAGGCAGAGTCA
α-SMA	Forward	CCCAGACATCAGGGAAGTAATGG
	Reverse	TCTATCGGATACTTCAGCGTCA
Collagen I	Forward	TAAGGGTCCCCAATGGTGAGA
	Reverse	GGGTCCCTCGACTCCTACAT
TGF-1β	Forward	AGCACAGTATGCAAGCCTCG
	Reverse	ATCTGTAATGTTGAACTGGGTGG
TIMP1	Forward	CACTCGGCTCTAGTGATACGG
	Reverse	CTTGGCCTTCTCGAACCCTTT
PINK1	Forward	TGCTGAAACTGCCTTCCTATCA
	Reverse	CCGCTAGTTGAACATACAGGATG

Parkin	Forward	GCTTCCGAAGGTGTGTCAG
	Reverse	CGGGCATTGCTCTCAGTCA
Beclin-1	Forward	ATGGAGGGGTCTAAGGCGTC
	Reverse	TGGGCTGTGGTAAGTAATGGA
GAPDH	Forward	GGTTGTCTCCTGCGACTTCA
	Reverse	TGGTCCAGGGTTTCTTACTCC
