# Supplementary figures and images for: Yixinjiedu Formula Attenuates Pressure Overload-Induced Cardiac Dysfunction by Suppressing Ferroptosis and Restoring Mitophagy via the PINK1/Parkin Axis
Source: Pharmaceuticals (Basel). 2026 Feb 25;19(3):360. doi: 10.3390/ph19030360 (PMC13028673; doi:10.3390/ph19030360)

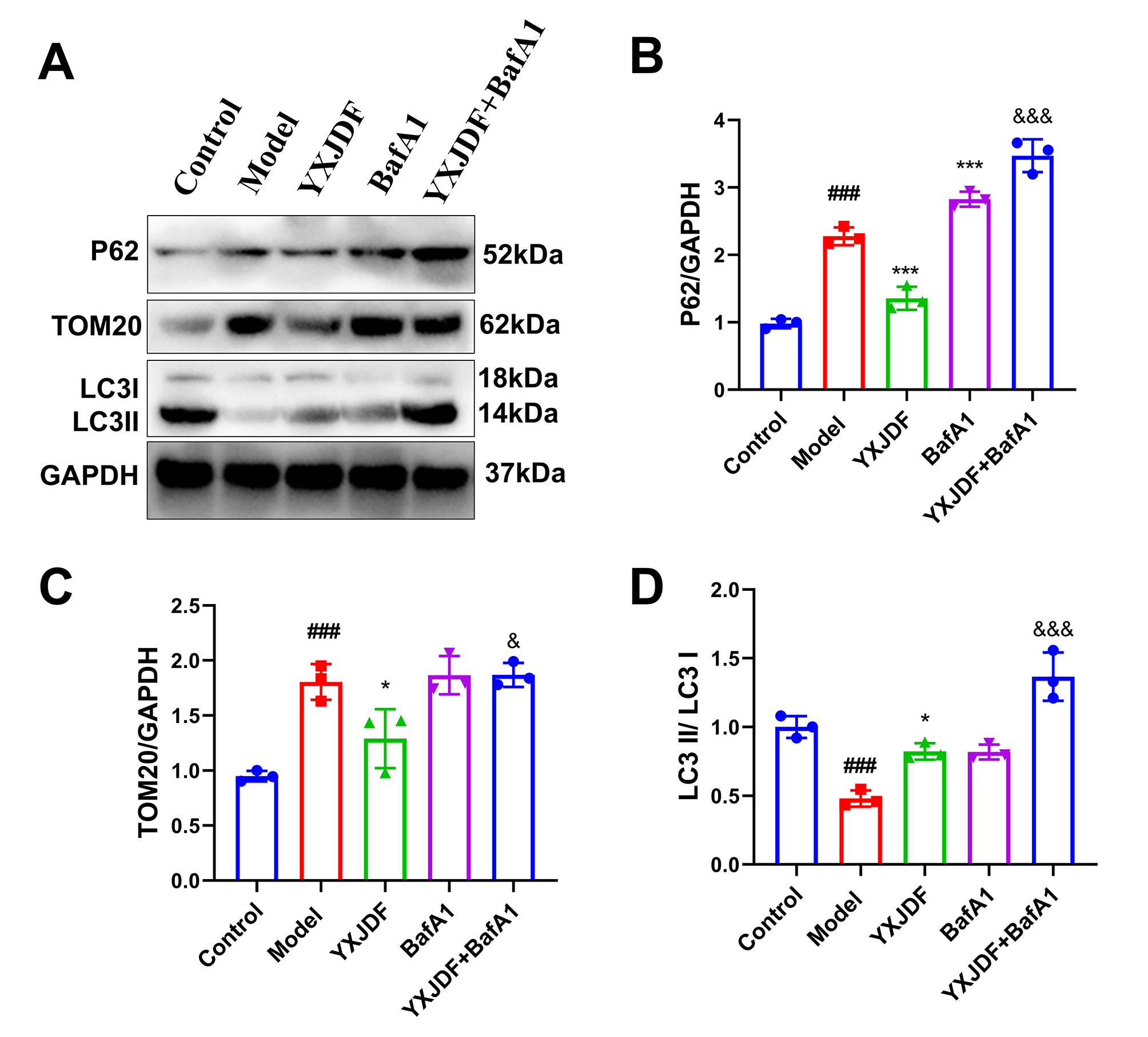

Supplement: Supplementary file 1 [file pharmaceuticals-19-00360-s001.zip › pharmaceuticals-4075594-Figure S1.tif]
